# Usefulness of Contrast-Enhanced Harmonic Endoscopic Ultrasonography for Diagnosis of Malignancy in Intraductal Papillary Mucinous Neoplasm

**DOI:** 10.3390/diagnostics12092141

**Published:** 2022-09-02

**Authors:** Yasunobu Yamashita, Yuki Kawaji, Toshio Shimokawa, Hirofumi Yamazaki, Takashi Tamura, Keiichi Hatamaru, Masahiro Itonaga, Reiko Ashida, Manabu Kawai, Masayuki Kitano

**Affiliations:** 1 Second Department of Internal Medicine, Wakayama Medical University, Wakayama 641-0012, Japan; 2 Clinical Study Support Center, Wakayama Medical University Hospital, Wakayama 641-8510, Japan; 3 Second Department of Surgery, Wakayama Medical University, Wakayama 641-8509, Japan

**Keywords:** intraductal papillary mucinous neoplasm, contrast-enhanced harmonic endoscopic ultrasonography, invasive intraductal papillary mucinous carcinoma, hypoenhancement pattern, mural nodule size

## Abstract

Intraductal papillary mucinous neoplasms (IPMNs) have a wide pathologic spectrum and it can be difficult to diagnose malignancy, including pathological grade. The aim of this study was to evaluate contrast-enhanced harmonic endoscopic ultrasonography (CH-EUS) for the diagnosis of malignant IPMN and IPMN-associated invasive carcinoma (invasive IPMC). From 5009 patients diagnosed with IPMN at Wakayama medical university between December 2009 and December 2021, 115 patients who underwent contrast-enhanced computed tomography (CE-CT), conventional EUS, CH-EUS, and surgical resection were enrolled. The detection of mural lesions was compared with pathological findings. Malignant IPMN and invasive IPMC were also assessed according to mural lesion size and vascularity on CH-EUS. CH-EUS and conventional EUS showed significantly higher accuracy than CE-CT in the detection of mural nodules (92%, 83%, and 72%, respectively) and diagnosis of malignant IPMN (75%, 73%, and 63%, respectively). An early wash-out pattern on CH-EUS was observed in significantly more patients with invasive IPMC than in those with low-, intermediate-, or high-grade dysplasia. When compared with CE-CT, CH-EUS was significantly more accurate for detecting mural nodules and more useful for diagnosing malignant IPMN. The vascular pattern on CH-EUS was also useful for diagnosing invasive IPMC.

## 1. Introduction

Intraductal papillary mucinous neoplasms (IPMNs) are pancreatic tumors that are accompanied by the dilatation of the excretory pancreatic ducts and mucous production due to papillary proliferation of ductal epithelium. The initial description of IPMN was published in 1982 [1]. IPMNs are the most common pancreatic cystic neoplasms, and their incidence has increased over time, from 0.3 per 100,000 person-years over 1984–1985 to 4.5 per 100,000 person-years over 2001–2005 [2]. Therefore, they are currently considered an important healthcare problem. 

IPMNs are slow-growing tumors that have a range of histological features from benign to malignant. Moreover, IPMNs are likely to occur in elderly patients, and resection requires invasive procedures, such as pancreaticoduodenectomy. It is therefore important to determine whether IPMNs are malignant, their pathological grades, and the prognosis for patients with IPMNs.

Endoscopic ultrasonography (EUS) is thought to be one of the most reliable and efficient diagnostic modalities for pancreatobiliary disease because of its superior spatial resolution compared with any other modality. In this respect, EUS is useful for detecting small lesions, such as mural nodules in IPMNs. IPMNs have a wide pathologic spectrum, and it can be difficult to differentiate malignant tumors, such as carcinoma in situ and invasive carcinoma, from benign tumors. The presence of a mural nodule in branch duct-type IPMNs of the pancreas is a marker of malignant transformation [3,4], and evaluation of mural nodules is therefore useful for diagnosing the degree of malignant IPMN. However, it is sometimes difficult to discriminate mural nodules from mucous clots on conventional EUS, and the assessment of mural lesion vascularity with contrast-enhanced harmonic EUS (CH-EUS) is useful for their differential diagnosis [5]. The vascular pattern on CH-EUS is also useful for differentiating malignant pancreatic cancer lesions from benign pancreatic lesions. In differential diagnosis, many previous reports showed pancreatic cancer to have a hypoenhancement pattern and benign pancreatic lesions, such as inflammatory masses and neuroendocrine tumors, were to have isoenhancement and hyperenhancenment patterns, respectively [6,7,8,9,10,11,12,13,14,15,16,17]. In a meta-analysis of CE-EUS for pancreatic solid masses, the pooled estimates of sensitivity, specificity, and AUC for diagnosis of pancreatic cancer were 93% (95% CI, 0.91–0.95), 80% (95% CI, 0.75–0.85), and 0.97, respectively [7]. The assessment of vascularity with CH-EUS is useful for detecting the pathological grade of malignancy in pancreatic neuroendocrine neoplasms and gastrointestinal stromal tumors (GIST) [18,19,20]. Ishikawa et al. reported that a hypoenhancement pattern on CH-EUS was an indicator of aggressive pancreatic neuroendocrine neoplasms, with sensitivity, specificity, positive predictive value, negative predictive value, and accuracy of 94.7%, 100%, 100%, 96.6%, and 97.9%, respectively [18]. Sakamoto et al. reported that irregular vessels on CH-EUS were an indicator of GIST malignancies with a sensitivity, specificity, and accuracy of 100%, 63%, and 83%, respectively [20]. Therefore, the vascularity assessment on CH-EUS may be useful for differentiating malignant IPMN from benign IPMN and diagnosing the pathological grade of malignant IPMN. The size of a mural nodule was reported to be an independent predictor of malignancy [3]. Moreover, the presence of mural lesions ≥5 mm in size is an important factor for making decisions on surgical intervention in the 2017 guidelines for IPMN [21]. Therefore, mural lesion size is useful for differentiating malignant IPMN from benign IPMN and diagnosing the pathological grade of malignant IPMN. In this study, we compared the diagnostic abilities of contrast-enhanced computed tomography (CE-CT), conventional EUS, and CH-EUS. We also evaluated the diagnostic ability of mural lesion size and vascular pattern on CH-EUS for differentiating between benign and malignant IPMNs, including identification of the pathological grade of malignancy.

## 2. Materials and Methods

### 2.1. Study Design

This retrospective observational study was performed at Wakayama Medical University Hospital. The study was approved by the ethics committee of Wakayama Medical University (No. 3012) and was performed in accordance with the ethical standards laid down in the 1964 Declaration of Helsinki. The primary outcomes were comparisons of the diagnostic ability of CE-CT, conventional EUS, and CH-EUS in the differential diagnosis between benign and malignant IPMN and the diagnosis of IPMN-associated invasive carcinoma (invasive IPMC), which was defined according to the presence of mural lesions. Secondary outcomes were evaluations of the differential diagnosis between benign and malignant IPMN and the diagnosis of invasive IPMC, for which mural size and vascular pattern on CH-EUS were employed. The detection rates for mural nodules on CE-CT, conventional EUS, and CH-EUS were also compared with those obtained using pathological findings.

### 2.2. Patients

From 5009 patients diagnosed with IPMN at Wakayama medical university between December 2009 and December 2021, 115 patients with IPMN who underwent CE-CT, conventional EUS, CH-EUS, and surgical resection were enrolled. The inclusion criteria were: age ≥ 20 years; non-main-duct type IPMN; and pathologically confirmed IPMN grade based on a surgical specimen. In this study, the patients with IPMN were divided into low- and intermediate-grade dysplasia (LGD/IGD), high-grade dysplasia (HGD), and invasive IPMC. Malignant IPMN or malignancy was defined as HGD and invasive IPMC.

### 2.3. EUS Procedure

Electronic radial-type or convex-type echoendoscopes (GF-UE260-AL5, GF-UCT260; Olympus, Tokyo, Japan) with an ultrasound processor (ALOKA ProSound SSD α-10; Aloka Co. Ltd., Tokyo, Japan; ARIETTA 850; FUJIFILM Healthcare, Tokyo, Japan) were used for the EUS procedures which were performed by several endosonographers who had at least 10 years of experience in EUS. The latter employed the extended pure harmonic detection method with the mechanical index set at 0.25. After reconstitution with 2 mL of sterile water for injection, 0.7 mL of the contrast agent (Sonazoid^®^; GE Healthcare Pharm, Tokyo, Japan) was administered through a peripheral vein for CH-EUS. Vascularity and vascular pattern were assessed by two endosonographers (who had at least 10 years EUS experience) after recording the EUS images. When independently gained assessments differed between the two reviewers, reevaluation was performed between the reviewers until an agreement was reached. 

### 2.4. Definitions

**Figure 1:**
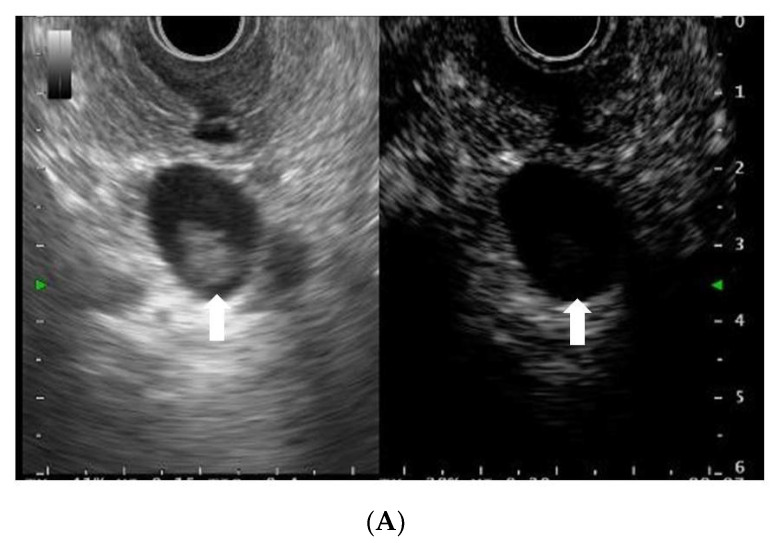

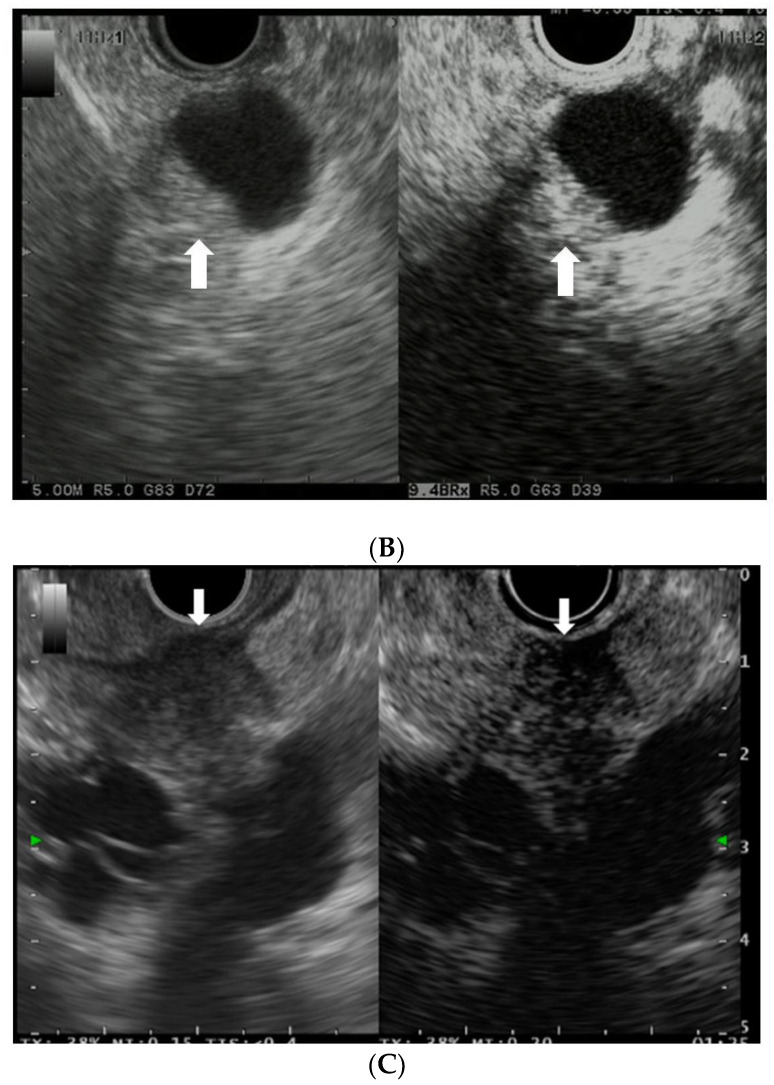
(**A**) Representative case of a mucus clot without vascularity. Conventional endoscopic ultrasonography (EUS; left) reveals an isoechoic mural lesion (arrow) in a cyst. Contrast-enhanced harmonic EUS (right) reveals a mural lesion (arrow) without vascularity. (**B**) Representative case of a mural nodule with vascularity. Conventional endoscopic ultrasonography (EUS; left) reveals an isoechoic mural lesion (arrow) in a cyst. Contrast-enhanced EUS (right) reveals a mural lesion (arrow) with vascularity. (**C**) Representative case of an invasive IPMC. Conventional endoscopic ultrasonography (EUS; left) reveals a hypoechoic lesion (arrow). Contrast-enhanced EUS (right) reveals a hypoenhancing lesion (arrow) due to early wash-out.

Lesions showing the presence of vascularity on CH-EUS were defined as mural nodules, whereas those with an absence of vascularity on CH-EUS were defined as mucus clots (Figure 1A,B). We defined malignant IPMN or invasive IPMC according to a mural lesion being detected on each modality. Vascular patterns on CH-EUS were classified as early wash-out and non-early wash-out. We defined an early wash-out pattern as a mural lesion exhibiting enhancement in the early phase and a rapid reduction of enhancement in the late phase on CH-EUS [22]. We defined malignant IPMN or invasive IPMC as a mural lesion showing an early wash-out pattern (Figure 1C).

### 2.5. Statistical Analysis

Statistical analysis was performed with JMP Pro version 13 (SAS Institute Inc., Cary, NC, USA). Fisher’s exact test for qualitative variables was used to compare categorical variables. For comparisons of diagnostic ability between the three imaging modalities, a Bonferroni-corrected *p*-value of <0.017 was considered to denote statistical significance according to a 0.05 decision threshold. The diagnostic accuracies of mural lesion size on each modality for determination of malignant IPMN and invasive IPMC were calculated from receiver operating characteristics (ROC) curves, with the maximum Youden index being used to determine cut-off points for which the sensitivity and specificity were calculated. Area under the ROC curve (AUROC) values were defined as low (0.5–0.7), moderate (0.7–0.9), or high accuracy (≥0.9) [23]. Differences were considered significant when the *p*-value was less than 0.05.

## 3. Results

The basic characteristics of the patients who underwent CH-EUS are shown in Table 1. No CH-EUS-associated adverse event was observed. The final pathological grade of malignancy in the 115 examined IPMNs were LGD/IGD (n = 31), HGD (n = 46), and invasive IPMC (n = 38). A mural lesion was detected on CE-CT, conventional EUS, and CH-EUS in 69, 103, and 93 cases, respectively. Using pathological diagnosis as the reference standard, the sensitivities, specificities, and accuracies for detection of mural nodules were 70%, 76%, and 72%, respectively, for CE-CT; 97%, 36%, and 83%, respectively, for conventional EUS; and 97%, 76%, and 92%, respectively, for CH-EUS (Table 2). Conventional EUS and CH-EUS were significantly superior to CE-CT for detecting mural nodules (*p* = 0.003 and *p* < 0.001, respectively). CH-EUS was also significantly superior to conventional EUS for detecting mural nodules (*p* = 0.009) and was the most accurate modality for detecting mural nodules.

The sensitivity, specificity, and accuracy for diagnosis of malignant IPMN including HGD and invasive IPMC (when the presence of a mural lesion was considered to indicate malignancy) were 65%, 55%, and 63%, respectively, for CE-CT; 93%, 19%, and 73%, respectively, for conventional EUS; and 88%, 39%, and 75%, respectively, for CH-EUS (Table 3). CH-EUS (75%) and conventional EUS (73%) showed significantly higher accuracy than CE-CT (63%) in the diagnosis of malignant IPMN (both *p* < 0.001). Adding CH-EUS to conventional EUS increased the specificity for malignant IPMN (including HGD and invasive IPMC) from 19% to 39% (Table 3). The sensitivity, specificity, and accuracy for diagnosis of invasive IPMC were 79%, 49%, and 59%, respectively, for CE-CT; 100%, 16%, and 43% for conventional EUS; and 100%, 29%, and 52% for CH-EUS when the presence of a mural lesion was considered to indicate invasive IPMC (Table 3). CH-EUS (52%) and CE-CT (59%) showed significantly higher accuracy than conventional EUS (43%) in the diagnosis of invasive IPMC (both *p* < 0.001; Table 3).

**Figure 2:**
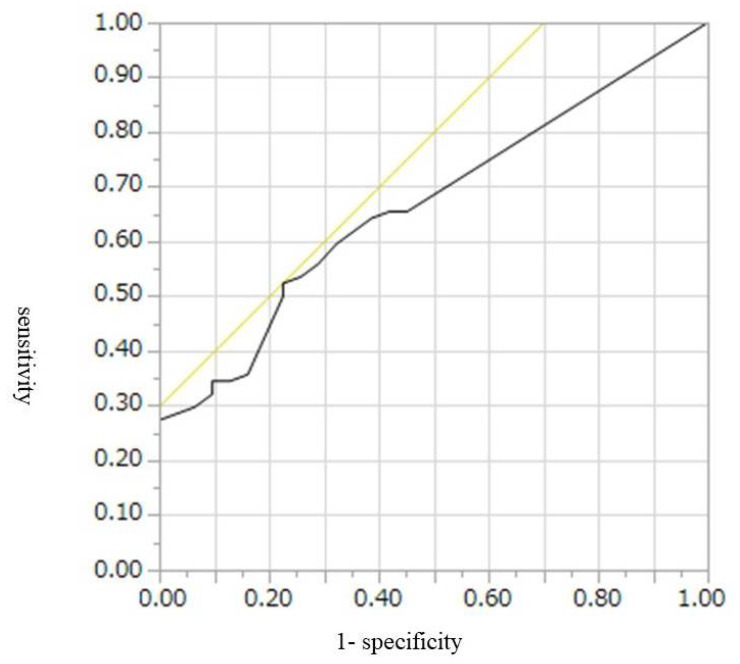
Receiver operating characteristics curve of the presence of mural lesions on contrast-enhanced CT for the diagnosis of malignant IPMN. CT, computed tomography; IPMN, intraductal papillary mucinous neoplasm.

**Figure 3:**
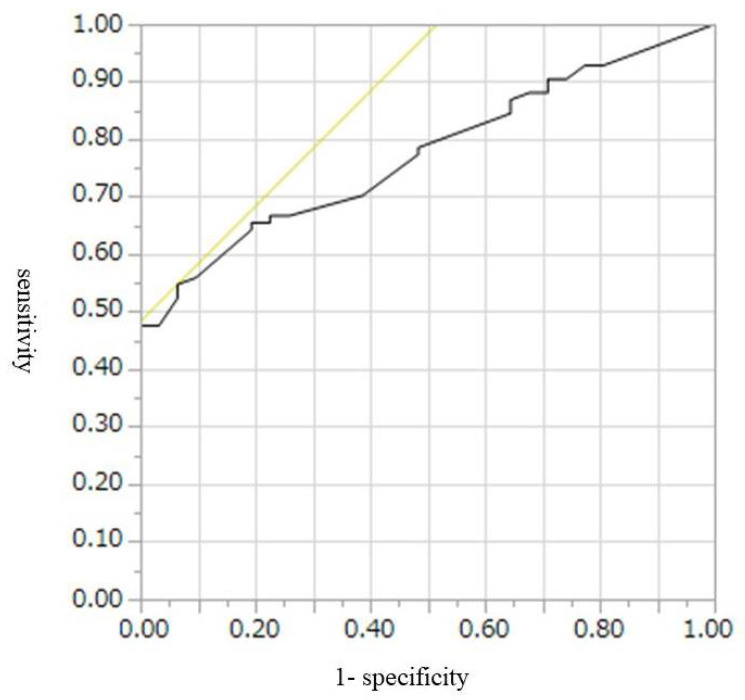
Receiver operating characteristics curve of the presence of mural lesions on conventional EUS for the diagnosis of malignant IPMN. EUS, endoscopic ultrasonography; IPMN, intraductal papillary mucinous neoplasm.

**Figure 4:**
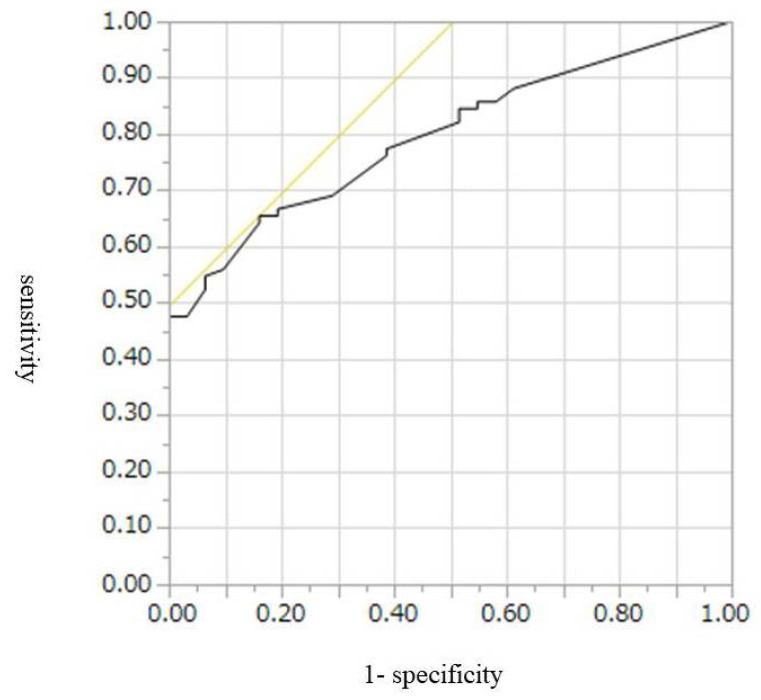
Receiver operating characteristics curve of the presence of mural lesions on CH-EUS for the diagnosis of malignant IPMN. CH-EUS, contrast-enhanced harmonic endoscopic ultrasonography; IPMN, intraductal papillary mucinous neoplasm.

**Figure 5:**
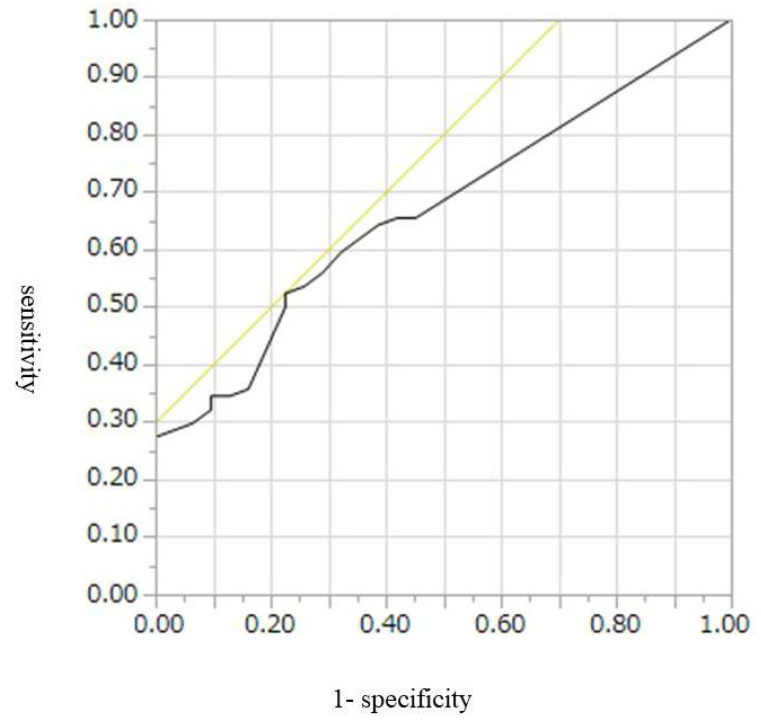
Receiver operating characteristics curve of the presence of mural lesions on contrast-enhanced CT for the diagnosis of invasive IPMC. CT, computed tomography; IPMC, intraductal papillary mucinous neoplasm-associated invasive carcinoma.

**Figure 6:**
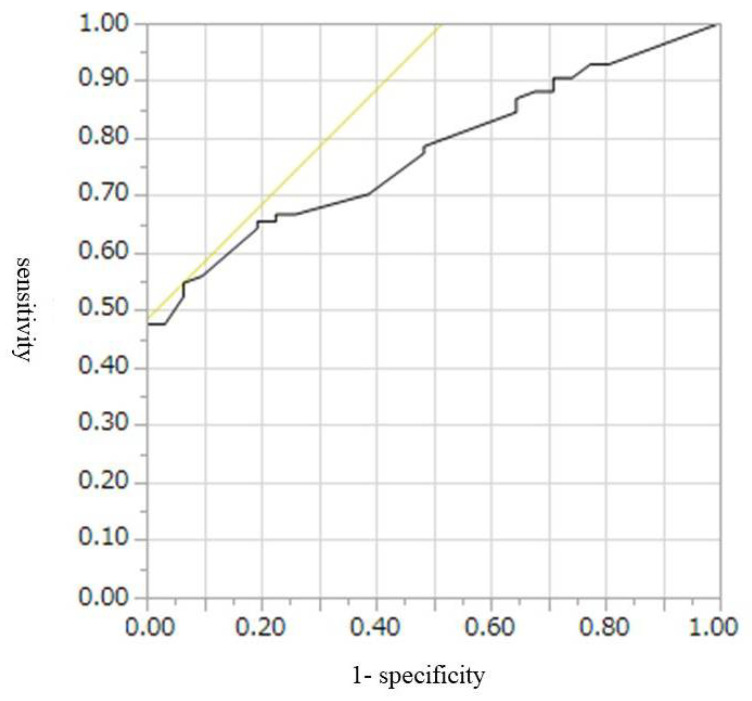
Receiver operating characteristics curve of the presence of mural lesions on conventional EUS for the diagnosis of invasive IPMC. EUS, endoscopic ultrasonography; IPMC, intraductal papillary mucinous neoplasm-associated invasive carcinoma.

**Figure 7:**
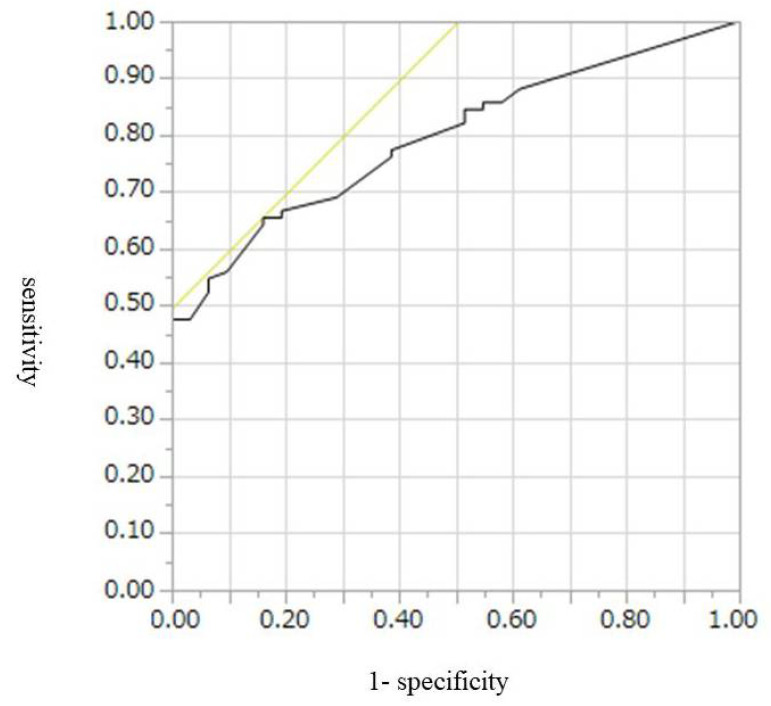
Receiver operating characteristics curve of the presence of mural lesions on CH-EUS for the diagnosis of invasive IPMC. CH-EUS, contrast-enhanced harmonic endoscopic ultrasonography; IPMC, intraductal papillary mucinous neoplasm-associated invasive carcinoma.

The cut-off values, AUROCs, sensitivities, and specificities for diagnosis of malignant IPMN, including HGD and invasive IPMC, were 9 mm, 0.67, 52%, and 74%, respectively, for CE-CT; 9.5 mm, 0.77, 55%, and 46%, respectively, for conventional EUS; and 7.8 mm, 0.80, 65%, and 84%, respectively, for CH-EUS (Table 4, Figure 2, Figure 3 and Figure 4). Conventional EUS and CH-EUS showed moderate accuracy for diagnosing malignant IPMN, including HGD and invasive IPMC. The cut-off values, AUROCs, sensitivities, and specificities for diagnosis of invasive IPMC were 7 mm, 0.74, 76%, and 65%, respectively, for CE-CT; 9 mm, 0.76, 76%, and 68%, respectively, for conventional EUS; and 7.8 mm, 0.82, 87%, and 65% for CH-EUS (Table 5, Figure 5, Figure 6 and Figure 7). CE-CT, conventional EUS, and CH-EUS showed moderate accuracy for diagnosing invasive IPMC.

An early wash-out pattern on CH-EUS was observed in 22 of 38 (58%) cases of invasive IPMC, but not in LGD/IGD (0/31) or HGD (0/46). An early wash-out pattern was observed in significantly more patients with invasive IPMC than in those with HGD or LGD/IGD (*p* < 0.001; Table 6). An early wash-out pattern was also observed in significantly more patients with malignant IPMN (including HGD and invasive IPMC) than in those with LGD/IGD (*p* < 0.001; Table 6).

## 4. Discussion

In this study, CH-EUS was performed in patients with IPMN who underwent surgical resection. CH-EUS was superior to conventional EUS and CE-CT for the detection of mural nodules and malignant IPMN, including HGD and invasive IPMC. Moreover, an early wash-out pattern on CH-EUS was useful for identifying invasive IPMC.

Currently, malignancy is diagnosed through the cytological examination of pancreatic juice obtained with endoscopic retrograde cholangiopancreatography [24], or via the measurement of biochemical and tumor markers in cyst fluid obtained with EUS-guided fine needle aspiration (EUS-FNA) [25,26,27,28]. However, the safety of these methods remains unclear because of the possibility of ERCP-related pancreatitis and peritoneal dissemination due to the leakage of cystic contents following EUS-FNA. Moreover, it remains difficult to diagnose the pathological grade of IPMNs using these procedures. However, the presence of a mural lesion in branch duct-type IPMNs of the pancreas is a marker of malignant transformation [2,3], and evaluation of mural lesions is therefore useful for diagnosing malignancy, including pathological grade.

Although the resolution of CE-CT has greatly improved with increases in the number of detectors and development of reconstructed images, CE-CT has limitations in the detection of small lesions. In comparison, EUS is thought to be one of the most reliable and efficient diagnostic modalities for small pancreatobiliary lesions because of its superior spatial resolution in comparison to any other modality. Indeed, in several reports, EUS was found to be superior to CT in terms of the ability to detect small pancreatic cancers [29,30,31,32]. In this study, we showed that CH-EUS and conventional EUS were significantly superior to CE-CT for detecting mural lesions. Moreover, CH-EUS was also significantly superior to conventional EUS because CH-EUS was useful for discriminating mural lesions from mucus clots, as documented in previous reports [5,33,34]. Fujita et al. also reported the utility of CH-EUS for the diagnosis of mural nodules in IPMN, and they included a larger number of IPMNs (427) in their study [34]. However, surgical resection was performed on only five cases for a suspected mural nodule and was not performed on cases suspected to be a mucous clot on CH-EUS. In contrast, in our study, surgical resection was performed on all 115 cases, and mural nodules in IPMN were assessed using only surgical resections. Although the presence of mural nodules ≥5 mm in size on conventional EUS is an important factor for making decisions on surgical intervention according to the 2017 guidelines for IPMN [21], we found that CH-EUS was superior to conventional EUS for detecting mural nodules, especially with regards to specificity because of misdiagnosis of mucus clots as mural nodules on conventional EUS. Therefore, we consider CH-EUS to be a necessary tool for determining the requirement for surgical resection of IPMN. CH-EUS is also advantageous when patients have contraindications to CE-CT contrast agents (e.g., because of renal failure or a contrast allergy), and it allows for dynamic and repeat examinations without exposing the patient to ionizing radiation.

We found that conventional EUS and CH-EUS were superior to CE-CT for the diagnosis of malignant IPMN, and that adding CH-EUS to conventional EUS increased the specificity for detecting malignant IPMN. Kamata et al. reported that CH-EUS was significantly superior to conventional EUS in terms of the specificity for identifying malignant IPMN when a mural lesion was defined as malignant IPMN [4]. The present study also showed higher specificity on CH-EUS than on conventional EUS. However, comparisons of our findings with those of Kamata et al. reveal that the specificity in our study (39%) was lower than that in the previous report (96%). There are two probable reasons for this discrepancy. First, the previous report included benign cystic lesions, such as serous cyst neoplasm and non-neoplastic cyst. When benign cystic lesions were excluded in a previous report, the specificity of CH-EUS was 44%, a result similar to ours. Second, the guidelines for surgical resection of IPMN were changed in an update. The previous report used the 2006 guidelines [33], whereas our study used the 2006, 2012, and 2017 guidelines [21,35,36]. The malignancy rate of resected cases in our study may be higher because more cases without mural lesion were followed, according to the strict selection for resection in the updated guidelines. Indeed, Hsiao et al. reported that rates of HGD and invasive IPMC on surgical resection were higher according to the 2012 guidelines than according to the 2006 guidelines [37]. In terms of the diagnosis of invasive IPMC, we found CE-CT to be superior to CH-EUS and conventional EUS in our study, although the accuracies of the three modalities were all low, ranging from 43% to 59%, when the presence of mural lesions was defined as invasive IPMC. Therefore, it is necessary to consider another method for diagnosis of invasive IPMC.

Yamamoto et al. evaluated the clinical impact of CH-EUS using the time intensity curve of the echo intensity of mural lesions in IPMN. They reported that the echo intensity change and echo intensity reduction rate of mural lesions were significantly higher in malignant IPMN than in LGD/IGD [22]. In particular, the echo intensity reduction rate of mural lesions was the most accurate parameter for diagnosing malignant IPMN. Therefore, the assessment of the vascular pattern of mural lesions is helpful for evaluating the pathological grade of malignancy. In our study, an early wash-out pattern in IPMC was shown in only invasive disease and was significantly more common in patients with invasive IPMC than in those with HGD or LGD/IGD. Thus, in clinical practice, the vascular pattern on CH-EUS may be valuable for the diagnosis of invasive IPMC.

Regarding the prognosis of patients with IPMN, Wada et al. reported that the 5-year survival rate was significantly higher in patients with non-invasive IPMC than in those with invasive IPMC (100% vs 46%, *p* < 0.001) [38]. Therefore, an early wash-out pattern on CH-EUS may predict a worse prognosis than a non-early wash-out pattern because all the early wash-out pattens observed in this study were found in invasive IPMC. Most pancreatic ductal carcinomas show a hypoenhancement pattern in the late phase on CH-EUS because of fibrous tissue with few vessels (as found in pathology) [9]. In the present study, the early wash-out pattern in mural lesions may reflect fibrous tissue with few vessels in invasive IPMC.

A meta-analysis suggested that mural nodule size has a considerable effect on the diagnosis of malignant IPMNs [39], although all the studies included in this meta-analysis used contrast-enhanced EUS to evaluate mural nodule size. This meta-analysis concluded that the use of CH-EUS seems to be mandatory because conventional EUS is less accurate [36]. In the present study, mural lesion size on CH-EUS had the highest ability to diagnose malignant IPMN and invasive IPMC. Our study proposes the use of CH-EUS to enable the more accurate diagnosis of mural nodules on EUS; therefore, CH-EUS should also be used for the assessment of mural lesion size.

This study has some limitations. First, this study was a single-center design and enrolled only a small number of cases. Further studies with a larger number of patients from multiple centers are required. Second, the calculation of specificity was limited because surgical resection was not performed in some IPMN cases, including benign ones. Third, HGD in sites other than mural nodules cannot be detected on CH-EUS, and HGD can be found in nodules in some cases.

In conclusion, CH-EUS is more accurate for detecting mural nodules and more useful for diagnosing malignant IPMN than CE-CT. The vascular pattern on CH-EUS is also useful for diagnosing invasive IPMC. CH-EUS may be useful for assessing the pathological grade of malignancy.

## Figures and Tables

**Table 1:** Characteristics of the Patients with IPMN.

	All (n = 115)	LGD/IGD (n = 31)	HGD (n = 46)	Invasive IPMC (n = 38)
Age in years, mean ± SD	70.5 ± 8.7	69.9 ± 7.4	70.6 ± 7.9	70.9 ± 10.9
Sex, male/female	63/52	19/12	27/19	17/21
IPMN size (mm)	29.1 ± 12.4	29.1 ± 11.2	29.9 ± 11.7	28.6 ± 14.6
MPD size (mm)	5.2 ± 2.4	4.3 ± 2.5	5.2 ± 2.4	5.9 ± 2.3
Stage (number)	-	-	0	IA (26); IIA (2); IIB (7); III (2); IV (1)

IPMN, intraductal papillary mucinous neoplasm; LGD, low-grade dysplasia; IGD, intermediate-grade dysplasia; HGD, high-grade dysplasia; IPMC, IPMN-associated invasive carcinoma; MPD, main pancreatic duct; CH-EUS, contrast-enhanced harmonic endoscopic ultrasonography; SD, standard deviation.

**Table 2:** Detection Abilities for Mural Nodules on CE-CT, EUS, and CH-EUS.

	CE-CT	Conventional EUS	CH-EUS
Sensitivity	70% (63/90)	97% (87/90)	97% (87/90)
Specificity	76% (19/25)	36% (9/25)	76% (19/25)
Accuracy	72% (83/115)	83% (96/115)	92% (106/115)

CE-CT, contrast-enhanced computed tomography; EUS, endoscopic ultrasonography; CH-EUS, contrast-enhanced harmonic endoscopic ultrasonography.

**Table 3:** Diagnostic Abilities for Malignancy and Invasive IPMC According to the Presence of Mural Lesion on CE-CT, EUS, and CH-EUS.

	CE-CT	Conventional EUS	CH-EUS
Sensitivity for malignancy	65% (55/84)	93% (78/84)	88% (74/84)
Specificity for malignancy	55% (17/31)	19% (6/31)	39% (12/31)
Accuracy for malignancy	63% (72/115)	73% (84/115)	75% (86/115)
	**CE-CT**	**Conventional EUS**	**CH-EUS**
Sensitivity for invasive IPMC	79% (30/38)	100% (38/38)	100% (38/38)
Specificity for invasive IPMC	49% (38/77)	16% (12/77)	29% (22/77)
Accuracy for invasive IPMC	59% (68/115)	43% (50/115)	52% (60/115)

IPMC, IPMN-associated invasive carcinoma; CE-CT, contrast-enhanced computed tomography; EUS, endoscopic ultrasonography; CH-EUS, contrast-enhanced harmonic endoscopic ultrasonography.

**Table 4:** Diagnostic Abilities for Malignancy According to the Size of Mural Lesion on CE-CT, Conventional EUS, and CH-EUS.

	Cut-Off Value (mm)	AUROC	Sensitivity (%)	Specificity (%)
CE-CT	9	0.67	52	74
Conventional EUS	9.5	0.77	55	46
CH-EUS	7.8	0.80	65	84

CE-CT, contrast-enhanced computed tomography; EUS, endoscopic ultrasonography; CH-EUS, contrast-enhanced harmonic endoscopic ultrasonography; AUROC, area under the receiver operating characteristics curve.

**Table 5:** Diagnostic Abilities for Invasive IPMC According to the Size of Mural Lesion on CE-CT, Conventional EUS, and CH-EUS.

	Cut-Off Value (mm)	AUROC	Sensitivity (%)	Specificity (%)
CE-CT	7	0.74	76	65
Conventional EUS	9	0.76	76	68
CH-EUS	7.8	0.82	87	65

IPMC, intraductal papillary mucinous carcinoma; CE-CT, contrast-enhanced computed tomography; EUS, endoscopic ultrasonography; CH-EUS, contrast-enhanced harmonic endoscopic ultrasonography; AUROC, area under the receiver operating characteristics curve.

**Table 6:** Diagnostic Abilities for Malignancy and Invasive IPMC According to an Early Wash-out Pattern on CH-EUS.

	Malignancy	Invasive IPMC
Sensitivity	26% (22/84)	58% (22/38)
Specificity	100% (31/31)	100% (77/77)
Accuracy	46% (53/115)	86% (99/115)

IPMC, intraductal papillary mucinous carcinoma; CE-CT, contrast-enhanced computed tomography; EUS, endoscopic ultrasonography; CH-EUS, contrast-enhanced harmonic endoscopic ultrasonography.

## Data Availability

No new data were created or analyzed in this study. Data sharing is not applicable to this article.
